# On-Demand Isolation and Manipulation of *C*. *elegans* by *In Vitro* Maskless Photopatterning

**DOI:** 10.1371/journal.pone.0145935

**Published:** 2016-01-05

**Authors:** C. Ryan Oliver, Eleni Gourgou, Daphne Bazopoulou, Nikos Chronis, A. John Hart

**Affiliations:** 1 Department of Mechanical Engineering, University of Michigan, Ann Arbor, MI, 48109, United States of America; 2 Department of Mechanical Engineering and Laboratory for Manufacturing and Productivity, Massachusetts Institute of Technology, Cambridge, MA, 02139, United States of America; 3 Department of Biomedical Engineering, University of Michigan, Ann Arbor, MI, 48109, United States of America; University of Illinois at Chicago, UNITED STATES

## Abstract

*Caenorhabditis elegans* (*C*. *elegans*) is a model organism for understanding aging and studying animal behavior. Microfluidic assay techniques have brought widespread advances in *C*. *elegans* research; however, traditional microfluidic assays such as those based on soft lithography require time-consuming design and fabrication cycles and offer limited flexibility in changing the geometric environment during experimentation. We present a technique for maskless photopatterning of a biocompatible hydrogel on an NGM (Agar) substrate, enabling dynamic manipulation of the *C*. *elegans* culture environment *in vitro*. Maskless photopatterning is performed using a projector-based microscope system largely built from off-the-shelf components. We demonstrate the capabilities of this technique by building micropillar arrays during *C*. *elegans* observation, by fabricating free-floating mechanisms that can be actuated by *C*. *elegans* motion, by using freehand drawing to isolate individual *C*. *elegans* in real time, and by patterning arrays of mazes for isolation and fitness testing of *C*. *elegans* populations. *In vitro* photopatterning enables rapid and flexible design of experiment geometry as well as real-time interaction between the researcher and the assay such as by sequential isolation of individual organisms. Future adoption of image analysis and machine learning techniques could be used to acquire large datasets and automatically adapt the assay geometry.

## Introduction

Capabilities for digital design and microfabrication of synthetic environments have advanced the study of model organisms such as *C*. *elegans*, *Drosophila sp*. and zebrafish. In the case of small organisms such as *C*. *elegans*, microfluidic assays have been used for immobilization and imaging, behavioral studies, laser microsurgery, and high throughput drug screening[1–4]. For example, microfluidic devices can enable modification of the synthetic environment during use via pressurization (e.g., valving) or by application of chemical, mechanical, or thermal stimuli to the specimen[3,5,6]. Despite advances in fabrication and capabilities of microfluidic assays, NGM plates remain a standard and widely accessible tool for *C*. *elegans* culture in biology and neuroscience laboratories. Even though microfluidic assays present elegant and effective capabilities, NGM plates have remained attractive as a culture substrate because nutrients can be provided on the surface, and these are inexpensive and easy to prepare within a standard cell culture plate.

Along with many advances in rapid prototyping techniques [7], maskless lithography presents a flexible means to create custom two-dimensional and three-dimensional microstructures on substrates for a variety of applications. For example, maskless lithography has been used, typically with a Digital Micromirror Device (DMD) projector, to fabricate microstructured surfaces [8], to pattern hydrogels [9,10], for in-flow lithography of microparticles [11], and for 3D printing of polymers and ceramics [12]. These techniques are being applied at a rapid pace, however to our knowledge the flexibility of maskless photopatterning has not been combined with *in vitro* environments. In the case of *C*. *elegans*, there is often a close connection between the design of the assay and the biologically relevant question that can be answered. For example, studies that aim to correlate learning and behavioral response to the plasticity of neuronal circuits could be aided by a tool that could select and isolate individuals that demonstrate unique learning capability, while maintaining viable culture conditions for long-term survival. It would be even more attractive to be able to modify the configuration of the assay in response to real-time events, as observed by the researcher, or as determined by an automated means. This is one of the needs we aim to address with our work.

In this paper, we demonstrate a new approach for experimentation with *C*. *elegans*, via photopatterning of hydrogel microstructures directly on NGM plates, which can be performed before or during culture of *C*. *elegans* on NGM. This approach is enabled by a custom maskless photopatterning system, and the identification of biocompatible hydrogel formulation that properly polymerizes on NGM plates. This method provides a rapid alternative to standard microfabrication methods for simple device geometries without external hardware, and adds flexibility to the workflow by enabling the researcher to incorporate new features in the assay based on dynamic observations as experimentation proceeds. We provide several demonstrations of this capability for dynamic modification of *C*. *elegans* assays during live optical imaging, and conclude by discussing its future potential.

## Materials and Methods

### Maskless photopatterning

**Fig 1:**
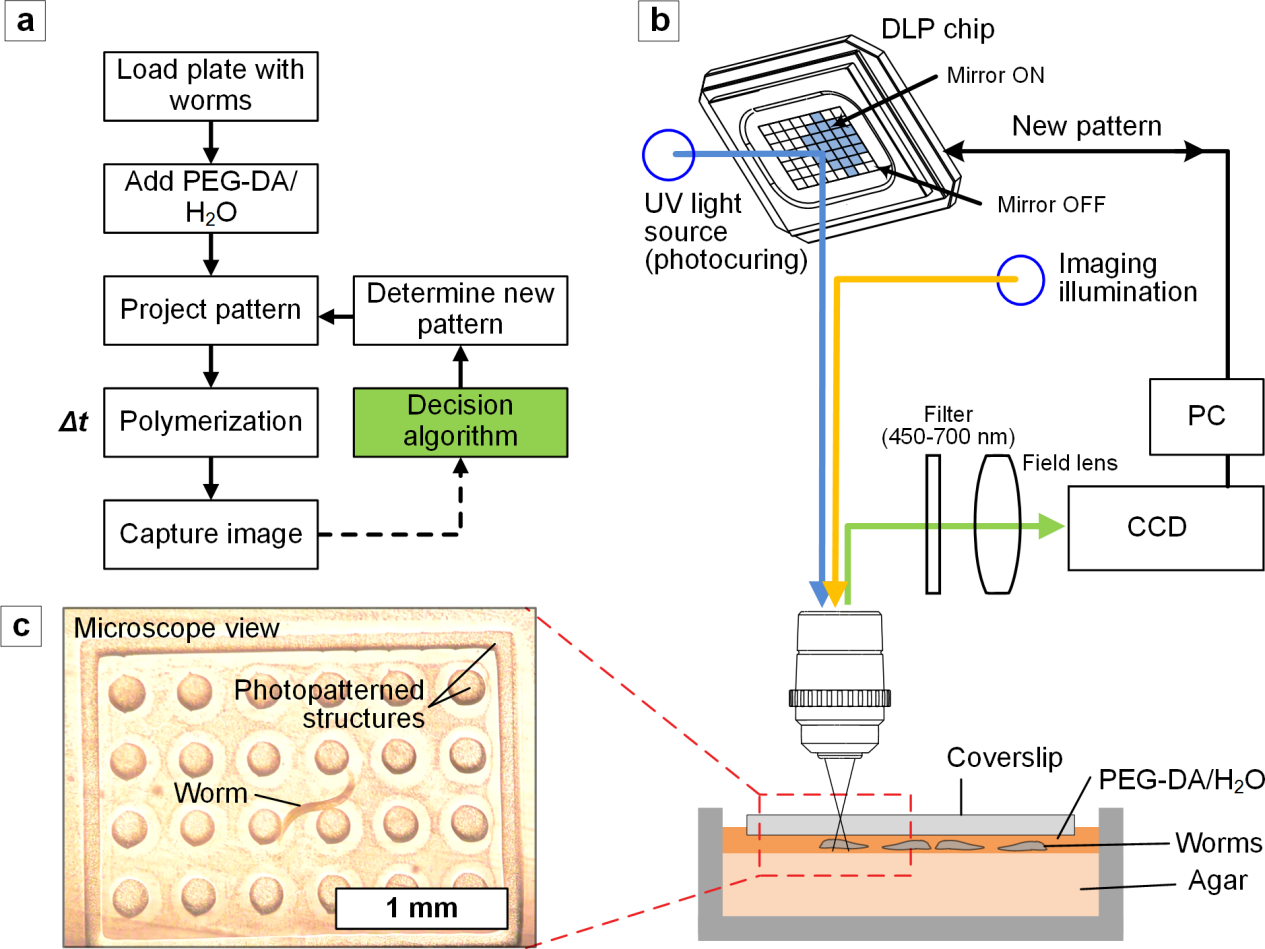
Dynamic photopatterning method and implementation. **(a)** Sequence, including real-time image capture and decision-based projection of the next fabrication step. **(b)** Schematic of the dynamic photopatterning system and configuration for patterning agar plates during *C*. *elegans* culture. Photo of the system is shown in **S1 Fig**. **(c)** An example of a framed micropillar array formed around a *C*. *elegans* worm *in situ*.

The maskless photopatterning system is designed to project an image onto the substrate to polymerize the hydrogel. It is composed of a mask generator (DMD, Wintech 4100), Nikon D5100 camera, an Ultra-violet (UV) light source (10W Mercury lamp, Dymax BlueWave 75) and a white light source (Zeiss CL1500) (**S1 Fig**). These are connected together into a functioning system using off-the-shelf optomechanical components which are detailed in **Table A** in **S1 File**. As shown in **Fig 1**, UV light is reflected off the DMD and is then filtered to 365 nm ± 30 nm. A 150 mm lens scales the image to match the aperture of the objective lens and is then projected through the objective lens (Mitutoyo 2X, 5X, 20X, 50X) onto the sample. The projection and sample are imaged using a Nikon D5100 SLR camera through the same optical train, via a beam splitter and a 200 mm tube lens.

We designed a custom 3D printed housing (**S1 Fig**) that positions a UV-capable DMD chip in place of the traditional mask. The housing accepts the fiber light guide and steers the collimated beam onto the DMD such that it reflects along the axis of the projection system. This is achieved by positioning the light guide along the diagonal of the DMD, as shown in **S1 Fig**. Finally, **S1 Fig** overlays the optical paths that represent the imaging, projection and illumination discussed previously onto the schematic of the system and relates them to a provided list of components used.

The system is controlled via LabVIEW and a National Instruments (NI-6008) DAQ card. The UV light is actuated by closing a resistance circuit using a 5V relay switch (SainSmart) controlled by LabVIEW. Exposure time is controlled either via LabVIEW or by a timer built into the BlueWave 75.

### Materials

The photopolymer used for the majority of experiments was 0.5% v/v photoinitiator 2-hydroxy-2methyl-1-phenyl-1-propanone (Darocur 1173, Sigma Aldrich) in poly(ethylene glycol) (400) diacrylate (PEG-DA, Sigma Aldrich) in 80% aqueous solution. NGM plates were prepared using standard methods outlined in Brenner et al [13]. The chemical structure for PEG-DA is shown in **S2 Fig**. Other photoinitiators used include Phenylbis(2,4,6-trimethylbenzoyl)-phosphine oxide (Sigma Aldrich) and a mixture of 0.01 mM Eosin Y (Sigma Aldrich) and 0.1% TEA (Sigma Aldrich) (See **Table B** in **S1 File**) [14].

### ATR-FTIR measurements of diffusion

Diffusion of the PEG-DA polymer into the NGM gel was measured by ATR-FTIR (Thermo Scientific Nicollet 6700, fitted with an ATR-FTIR sampling accessory with a Germanium crystal). A 500 μm thick sample of NGM was placed on top of the crystal and 10 μl of PEG-DA was pipetted onto the surface of the NGM. A baseline spectrum was taken and remeasured periodically over 20 minutes (**S3 Fig**). Bands were measured against the location of known chemical expressions. Because the system identifies molecules at a specific depth in the sample we assume that the development of a band is due to diffusion [15].

### Survival assay

Synchronous populations of nematodes were established by allowing 20 adult hermaphrodites to lay eggs for a limited time interval (4–5 h) on NGM plates seeded with *Escherichia coli* OP50. Progeny were grown through the L4 larval stage, and then transferred to fresh PEG-DA (cured) coated (20% PEG-DA in water) NGM-seeded plates at groups of 10–20 nematodes per plate and kept at 20°C. The first day of adulthood was used as day 0. Animals were transferred to fresh plates every 2–4 days thereafter and were examined every day for pharyngeal pumping and movement in response to a gentle tap with a thin platinum wire or pipet tip, until death. Lifespan was defined as the number of days between day 0 and the last day on which the worm was scored as alive. Worms that died due to internally hatched eggs, desiccation, an extruded gonad or crawling on the edge of the plates, were incorporated into the date set as censored (**S5 Fig**).

### Statistical analysis

We used the Origin software package (OriginLab Corporation, Massachusetts, USA), to carry out statistical analysis and to determine lifespan values. The Kaplan Meier estimator was used to evaluate differences between survivals and determine the p-values.

### *C*. *elegans* tracking using image processing

Custom Python/Matlab code built using the OpenCV toolkit was written to identify the worm in each frame. Image differencing between frames was used to identify the motion of the work. Then blob detection was used to generate an array of possible worm locations. Moments calculated from the blobs generated the centroid of the worm’s position. This was done for each frame and the position, velocity and acceleration of the worm were derived from the resulting data set.

## Results

### Maskless photopatterning of PEG-DA microstructures on agar plates

We fabricate microstructured assays made of a common hydrogel (PEG-DA, Poly(ethylene glycol) Diacrylate) on NGM plates as described in Methods. The key advance over previous techniques using conventional microfabrication[7,16–20] is the use of a maskless photopatterning system, coupled with custom image analysis software, which enables real-time photpatterning of the hydrogel-containing medium during live *C*. *elegans* assays. The photopatterning system, as shown in **Fig 1**, uses a digital micromirror device (DMD) to generate diffraction limited projections of an image through a microscope[11,21–24]. An example of the resulting structures formed around a *C*. *elegans* nematode, as seen through the CCD, is shown in **Fig 1B**. S1 File shows detailed schematics of the system and includes a list of all components.

Maskless photopatterning of an assay involves three main steps. First, a NGM plate without a bacterial lawn is procured and then dried under flowing nitrogen for 30 seconds. No NGM plates containing a bacterial lawn were used in tests because the lawn prevented adhesion of the polymerized microstructures to the plate. Drying the surface under nitrogen was intended to standardize the moisture content at the top surface of the plate. Next, 5 μL of the photopolymer (PEG-DA/H_2_O/Photoinitiator), optionally containing worms, is dispensed onto the plate using a pipette. When it is desirable to control the height of the photopatterned structures, a cover slip is used in conjunction with polystyrene or glass beads to define the gap between the NGM and the cover. Surface tension draws the cover downward into contact with the beads, which are first placed around the periphery of the target area, defining the gap by the bead diameter. Finally, the substrate is inserted into the microscope and the pattern is projected onto the plate, creating the polymerized features and simultaneously bonding it to the agar. The cover slip, if used, is then removed from the plate. When worms are not present, the uncured PEG-DA can be rinsed away with buffer solution while the photopatterned structures remain adhered to the NGM. Because photopatterning can be performed with in situ imaging during live culture, additional exposures can fabricate new features without loss of registration; these can be stitched to span a larger area, and as shown later can be created in response to dynamic events occurring during the experiment.

### Materials selection and viability

Critical to the method is the use of a biocompatible polymer that is photocurable and adheres well to a substrate suitable for *C*. *elegans* culture. Before selecting PEG-DA/H_2_O/Darocur 113 on NGM for this study, we investigated several bio-adhesives and monomers including PEG-DA[10,19], Pluronic-F127[25], Methacrylate and PDMS. We chose PEG-DA from these options for its ease of polymerization, biocompatibility, and hydrogel properties (e.g. oxygen diffusion, low elastic modulus)[26]. Specifically, the other materials did not readily polymerize of maintain biocompatible conditions. In addition, the photoinitiator must be selected carefully based on its wavelength sensitivity and biocompatibility. Biocompatibility can be affected by the formation of free radicals during exposure to light or by hydrophobicity of the photoinitiator in use. We evaluated three photoinitiators: 2-hydroxy-2-methyl-1-phenyl-1-propanone (Darocur 1173), Phenylbis (2,4,6-trimethylbenzoyl)-phosphine oxide, and a combination of 0.01 mM Eosin Y and 0.1% TEA (Triethanolamine)[14]. As noted in S1 File, we chose to proceed with Darocur 1173, which had a suitable combination of biocompatibility, adhesion to NGM, and polymerization rate (See **Table B** in **S1 File**).

### Survival of *C*. *elegans*

Before performing live worm assays, it was essential to evaluate the viability of the worms in the presence of the un-cured PEG-DA. We ran survival assays testing various concentrations of PEG-DA solution, as described in S1 Data. We found that 20% PEG-DA in dI-H_2_O, does not result in signs of stress such as reduced motility or dehydration (**S4 Fig**) nor does cured PEG-DA affect worm lifespan (**S5 Fig)**. Therefore, we used this formulation for the following experiments to demonstrate the versatility of the dynamic photopatterning technique.

### Resolution of maskless photopatterning on NGM

**Fig 2:**
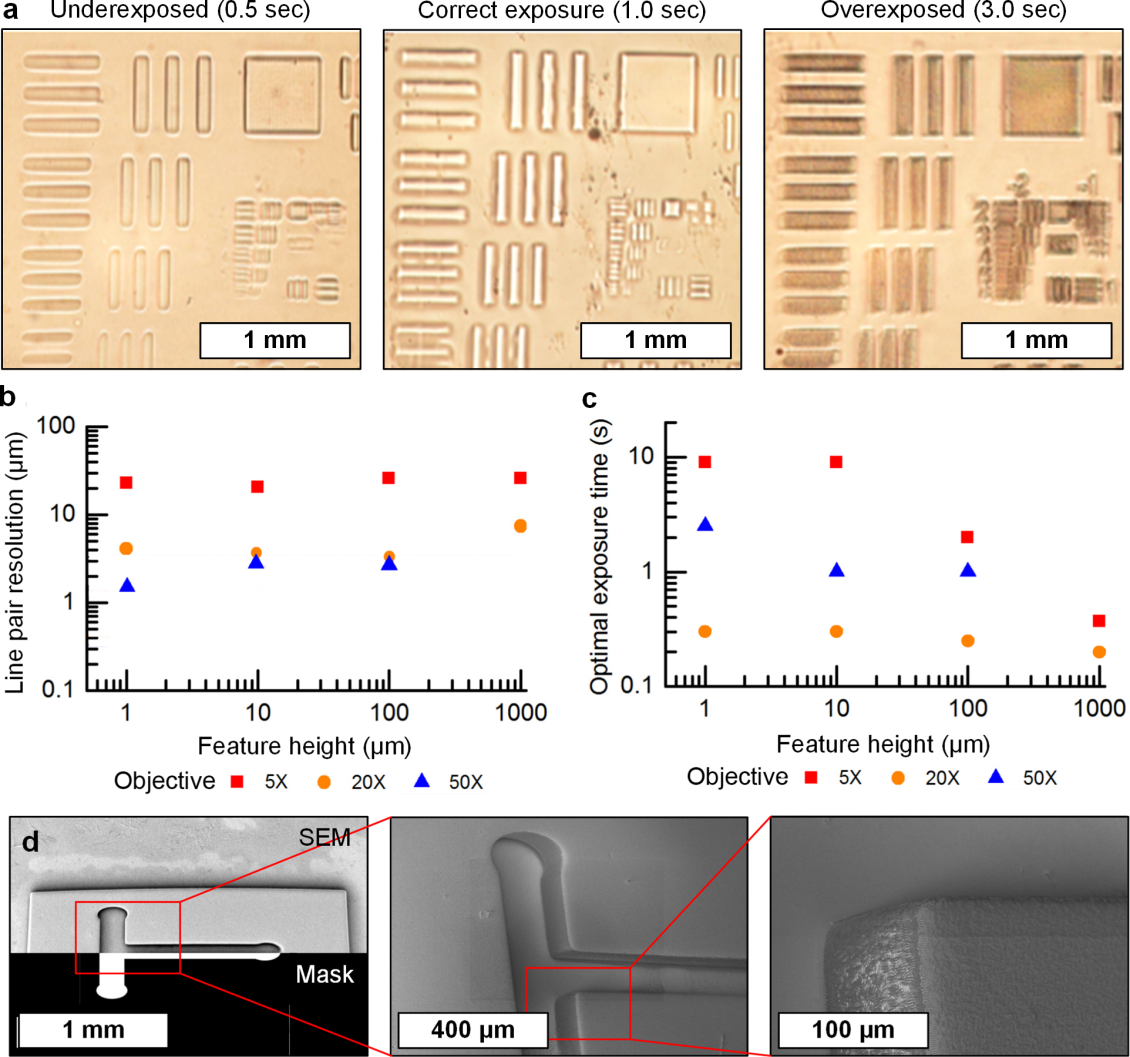
Resolution and exposure data for PEG-DA on agar. **(a)** Optical images of fabricated test pattern (USAF 1951 pattern) used to determine line pair resolution, with examples (from left to right) of under-exposed, properly exposed, and over-exposed results. **(b)** Relationship between line pair resolution and feature height for different objective magnifications indicated in the legend. **(c)** Relationship between optimal exposure time and feature height, determined using smallest line pair fabricated in each case. **(d)** SEM images of T-mazes as used in **Fig 6**, including (from left to right) the full maze in the upper half and the projected mask overlaid in the lower half, the sub-image shows one leg of the maze, and the right sub-image shows the corner of a wall feature.

A standard (USAF-1951) optical test pattern was used to determine the minimum line pair resolution (**Fig 2A and 2B**) of the photopatterning process. The line pair resolution represents the minimum distance between two parallel lines that can still be distinguished with a contrast greater than 30%. The exposure time was controlled using a shutter with a mechanically limited minimum exposure time of 50 ms. The duration of the exposure determines the thickness of the cured PEG-DA layer, which is in turn limited by the gap between the NGM and cover. The optimal exposure time for each objective and thickness was chosen to correspond to the highest resolution line pair measured, with five points taken on each sample. However, this cannot serve as a measure of the hydrogel stiffness in response to exposure conditions.

We find that photopatterning line pair resolution is dependent on feature height, for thick (10–100 μm) films, resolution is ~3 μm using a 50X objective lens and ~23 μm using a 5X objective, for thin (1 μm) films resolution increases to 0.7 μm and 1 μm for the 50X and 20X respectively. Moreover, as shown in **Fig 2C**, we find an inverse relationship between exposure time and the height of the polymerized feature. This may seem counterintuitive, yet is likely caused by oxygen diffusion through the NGM which inhibits polymerization at the hydrogel/NGM interface [26]. As a result, thinner PEG-DA films between the cover glass and NGM have slower polymerization kinetics. Control over in-plane feature geometry is shown by SEM images of an example T-maze structure (**Fig 2D**), compared to the projected mask. Using a 5X objective lens for projection, the polymerized features of 50 μm height have corner radii of approximately 10 μm.

Importantly, we found that polymerized features smaller than 50 μm would break off from the agar upon rinsing with buffer (see Methods). We assigned this as the minimum positive (i.e., raised) feature size we could build on NGM plates, having an aspect ratio of ~2:1which is still significantly smaller than the worm body length (~1 mm), and comparable to the body width. Negative features (i.e. channels) can of course approach the line pair resolution and therefore be much smaller than the width of young worms (~15 μm diameter for L1). Adhesion of the PEG-DA to NGM is governed by the diffusion at the interface; using attenuated total reflectance Fourier transform infrared spectroscopy (ATR-FTIR) we found that PEG-DA diffuses into the agar substrate (**S3 Fig**). After five minutes of diffusion, exposure to light creates a bond between the two interfaces. Without allowing time for diffusion samples exposed to light did not adhere to the NGM plate.

### *In vitro* maskless photopatterning during *C*. *elegans* culture

#### Manipulation of *C*. *elegans* by dynamic confinement

**Fig 3:**
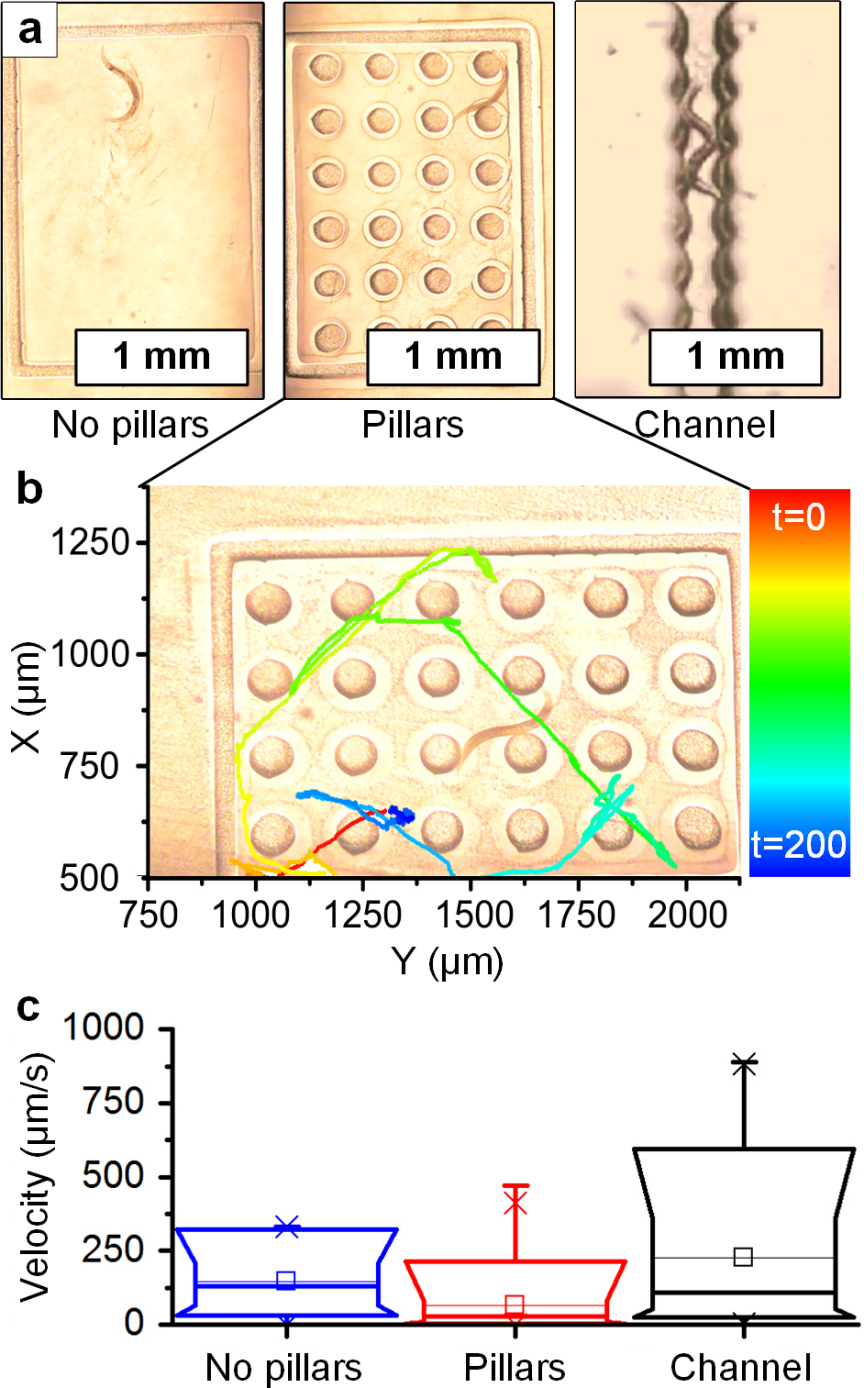
*In situ* photopolymerization of microstructures resulting in physical confinement of a *C*. *elegans*. **(a)** Three exemplary assays built sequentially (from left to right): an open frame, an array of micropillars (100 μm diameter), and a rippled microchannel (approx. 200 μm wide). **(b)** Tracking of the worm motion over a time period of 200s, within the pillar array. **(c)** Box-whisker plots of velocity in each configuration, showing that sequential confinement increases the maximum velocity at which the worm pushes against the surface of features. **S1 Video** shows the experiment.

To demonstrate the capability to build and modify locomotive *C*. *elegans* assays *in situ* we first designed experiments for comparison to previous studies [27] that used pre-fabricated PDMS devices. We chose to study whether *in situ* photofabrication of micropillars around swimming *C*. *elegans* would influence the worms’ swimming velocity and behavior. Starting with NGM plates loaded with worms, a single worm was isolated from the population by a single exposure (1s) using the lithography system to create a frame (5×3 mm, 100 μm depth), as shown in **Fig 3A**. Four seconds later, using a second exposure, we built an array of cylindrical micropillars within the frame (100 μm diameter, 100 μm center-center distance). The subsequent path of the worm in the pillars for 200 seconds is shown in **Fig 3B**. After the second assay was completed, a third exposure confined the worm within a corrugated microchannel approximately 100 μm wide. This experiment is also shown in **S1 Video**.

Going from the open frame, to the micropillar array, to the rippled microchannel, the worm’s maximum speed increased 270% from 330 μm/s to 890μm/sec. By comparing the maximum speed of the worm after each change was made to its environment (**Fig 3C**), we conclude that the worm swims faster when confined, which corroborates previous findings by Majmudar et al [28] using PDMS micropillars. However, distinct from prior work, our experiment demonstrates how dynamic photopatterning can be used to isolate individual worms from a population, and fabricate microstructures in real-time based on a specific goal that may be refined with each step of the experiment and intermediate observation. Moreover, our method eliminates the need to load worms into the device one-by-one and sort them into individual chambers, because the chamber can be fabricated *in situ* around each worm. We must note that, in the experiment above, the worm was able to partially swim between the left side of the photopatterned frame and the substrate, because the left side of the frame was not fully polymerized from the cover slip to the NGM substrate. Based on intensity measurements of the projection, we conclude this is caused by uneven illumination during the photopatterning step. This is caused by the Gaussian shape of the light intensity from the light source. This could be remedied in the future by placing two lens arrays facing in opposing direction between the light source and the DLP.

### Free-floating structure and mechanical elements

Dynamic photopatterning can also build free-floating features that can move in response to forces exerted by the swimming worms and/or by the fluid flow. Free-floating features cannot be fabricated in traditional PDMS-based devices, except via complex multilayer processing and/or specialized assembly methods[29]. The maskless photopatterning technique enables *in situ* multi-layer fabrication by adjusting the placement of the focal plane, and by selecting the exposure time such that polymerization of certain features occurs without attachment to the agar plate or the cover glass.

**Fig 4:**
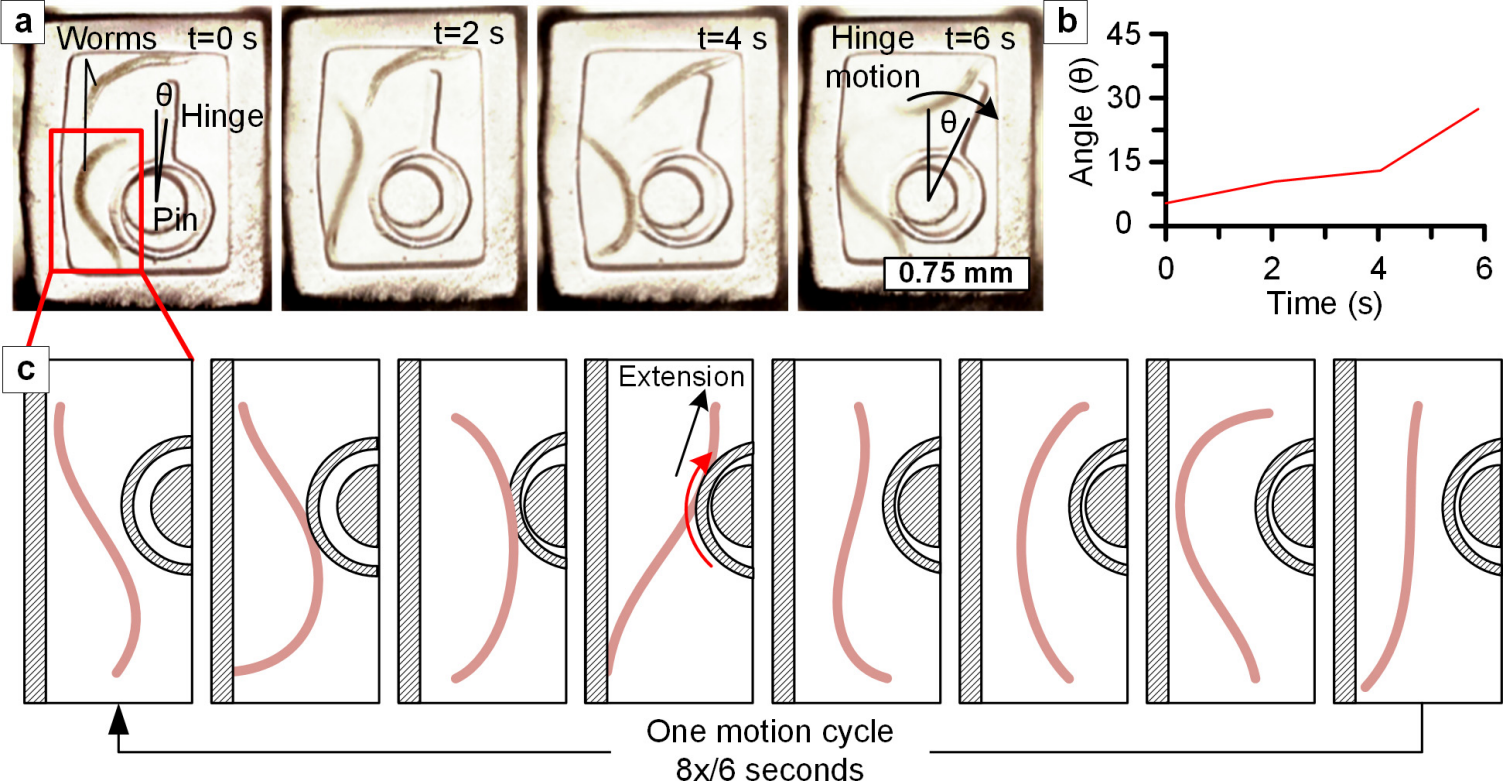
Interaction between worms and a simple hinge-pin mechanism fabricated within a millimeter-scale area. **(a)** Sequential frames show the lower worm contacting the hinge and extending its body, causing the hinge to rotate around the encapsulated pin. **(b)** Angle of the hinge with respect to vertical position plotted against time. Over six seconds the hinge rotates from 6° to 27°. **(c)** Schematics of worm motion as observed in the experiment, which is also shown in **S2 Video**. When the worm contacts two points spanning from the frame to the hinge, it extends its body, exerting force on the hinge and causing it to rotate. The motion cycle is repeated, rotating the hinge clockwise a small amount with each cycle.

In **Fig 4**, we show a lever (free-floating) fabricated around a pin (anchored to the NGM). An experiment was performed with two *C*. *elegans* worms confined within a frame surrounding this mechanism, and we observed one worm dynamically interacting with the lever (**Fig 4 and S2 Video**). The motion of the worm was periodic and touch reactive, as shown in the schematic of **Fig 4B**. The worm repeatedly wedged its body between the outer wall and the hinge. Once in that position, it straightened its body, pushing the hinge up and around the pin. Using this method, a variety of shapes and mechanisms could be fabricated within the culture environment, such as one-way gates, floating microparticles, movable isolation chambers and gears.

### Free-hand real-time drawing of features within the assay

**Fig 5:**
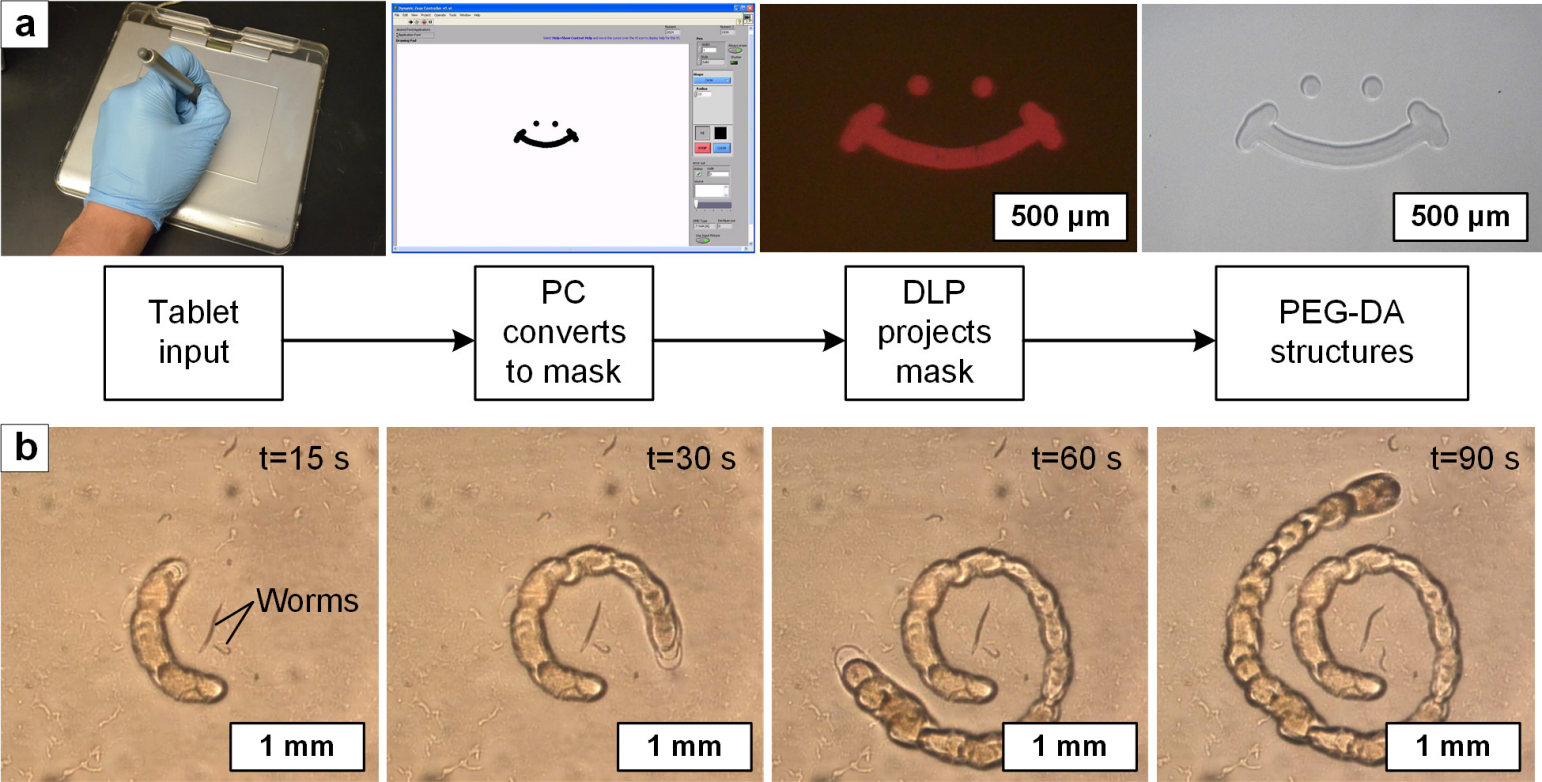
Tablet-based method for real-time assay modification by hand drawing. **(a)** Schematic of sequence, resulting in projection of manual tablet input to create PEG-DA features on the substrate. In this case, the scale of the hand drawing is reduced 50X. **(b)** Video frames of the photopatterning of a series of dots drawn by the researcher to confine a worm inside a spiral frame (**S3 Video**). The system projects each feature drawn by the researcher after a delay of 0.25 seconds.

In addition to building assays by projecting pre-defined geometric patterns, the researcher can generate freeform input using a tablet (**Fig 5**), resulting in real-time modification of the assay. This input is scaled and translated to the light pattern projected onto the NGM. Using a custom-built LabVIEW program, the latency, defined as the time between when the pen touches the pad and the projection of the corresponding light pixel, is approximately 250 ms. Once the pen touches the tablet, the computer converts the signal into a projected image. After this, it takes approximately 1 second to photopolymerize the PEG-DA.

A straightforward use of this capability is to manually isolate selected nematodes by freehand drawing, in registry with real-time video taken through the microscope system. The progressive isolation of a worm in a millimeter-scale spiral, fabricated incrementally by automated photopolymerization upon tablet input, is shown in **Fig 5B**. Here we show that local isolation can be achieved by maskless control of the illumination pattern rather than by repositioning the assay under a single point light source. Therefore, freeform patterns can be created, and individual organisms can be addressed without requirement for motion of the stage, which may be limited in rate or direction.

### Dynamic maze fabrication

In *C*. *elegans* population studies, locating individuals with exceptional fitness is a necessary first step to then selectively breed desired traits. Therefore, we last chose to demonstrate how individuals can be tracked in custom-made mazes to determine their ability to locate food.

**Fig 6:**
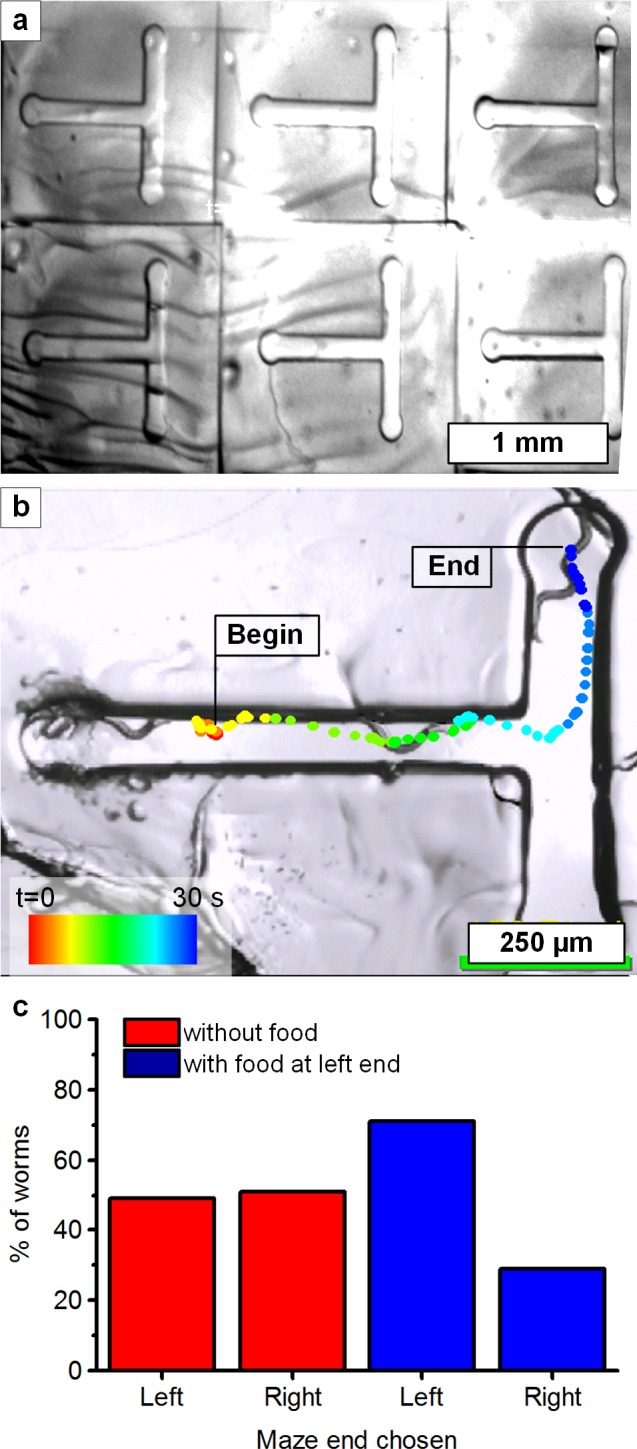
Fabrication of maze assays as a test case of *C*. *elegans* decision-making behavior. **(a)** Array of T-shaped mazes. The observed ripples are part of the agar surface. **(b)** T-shaped PEG-DA microchannel, with dot sequence indicating the centroid position of the worm during a 30 second period. **(c)** Percentage of worms that ended at each leg of the maze after being inserted into the entrance of the maze. See **S4 Video** for video of a nematode solving a maze.

Thirty-eight adult hermaphrodite worms were placed into the entrances of an array of T-shaped mazes (130 μm depth), first built by dynamic lithography. The motion of each worm was tracked until 80% of its body was inside a circular region at either terminal end of the maze (**Fig 6A**). Without the presence of food in the maze, the worms chose the left or right ends with equal probability (**Fig 6B**)[2]. In the next experiment, we placed food (*Escherichia coli* OP50, in liquid form) on the left end of each maze and we recorded the choice made by forty-two adult hermaphrodites. We found that the worms would choose the leg containing the food almost twice as often as the leg without food (**Fig 6B**). While this response could be due to the worms sensing food it could also be due to moisture or medium wicking along or diffusing through the PEG-DA gel or NGM surface.

Because these experiments did not include using a cover over the mazes, we also observed some worms crawling upward and out of the maze; this has also been seen in prior studies using PDMS fabricated mazes[2,30]. Worms that climbed their way out of the maze, even in initial stages, were not put back into the maze and were excluded from the study, so as to eliminate the possibility of them being already accustomed to the structure. In ongoing research we are studying how to select worm phenotypes based on their problem solving capability in progressively challenging mazes, by incrementally building the next stage of each maze based on analysis of the worm’s behavior in the prior stage.

## Discussion

We show that maskless photopatterning of PEG-DA enables rapid fabrication of simple microstructured assays on NGM plates, and *in situ* modification of the environment during live culture. This presents a significant change to the workflow of the researcher, who can now perform a controlled assay directly on NGM culture plates, rather than using lithographic and replica-based methods to fabricate devices in advance. This, in our opinion enables novel experimental designs, by enabling simultaneous rather than sequential assay fabrication and experimentation. Moreover, our method enables modification of assays during experimentation to isolate, direct or remove specific nematodes. The maskless photopatterning process is rapid, taking approximately 15 minutes to perform a complete experiment from design to data collection when all supplies have been prepared in advance.

The maskless technique can pattern assays with feature sizes in the 1–10 μm range, over a 100 mm diameter culture plate by using the motorized stage to move the plate underneath the optical system. The useful resolution depends on the assay design. For most experiments that involved confinement we found the 5X objective to be most useful, which can pattern negative features at a height of 100 μm with a resolution of 26 μm. For features fabricated prior to nematodes being added to the NGM plate, higher magnification can be used to generate features such as microtextures and obstacles on the surface of the NGM plate, which influence the *in vitro* environment but do not necessarily confine the worm.

Finally, maskless photopatterning on NGM plates enables a simple method of loading a worm into the device because the device is built around the worm already on the plate. However, our method cannot yet realize some of the more sophisticated functions of other systems such as localized delivery of chemicals, automated imaging, and valving via pressurization.

We acknowledge that the results presented here simply confirm conclusions made using similar experimental designs, albeit with traditional assay fabrication methods. As a result, our findings confirm these results yet illustrate the additional challenges that our method presents, especially with regard to material selection, analysis and interpretation of the collected data to ensure variations between experimental repetitions are accounted for. It should also be noted that additional study would be required to confirm the use of this method for long term studies, including investigation of biochemical effects due to the photoinitiator. Furthermore, we did not evaluate the possible biochemical impact of UV exposure as the pulse used here is significantly shorter than the irradiation time during transgene integration. Also, it is possible to use a green light (520 nm) platform instead because of DLP technology and a green light photoinitiator that became available since we began the present study. That said, the flexible and dynamic nature of our method, if combined with advanced software and more complex assay designs, could be a useful tool in a broader spectrum of behavioral studies. For example, free-floating microstructures could be used to observe how *C*. *elegans* interact with structures found while swimming, and potentially how they can be trained by repeating tactile triggers. Moreover, the additional capability of dynamic feedback can be used to incorporate new features into an assay based on real-time automated analysis, or to encapsulate a phenotype of interest for further processing. Because of its use of an open NGM plate, our method could be effectively combined with real time worm tracking systems and fluorescent imaging of selected neurons, in order to monitor which neurons and neuronal circuits participate in activities such as solving a maze, seeking food, and readjusting locomotion routes [31–33]. Progress in materials synthesis will also be important; for example, a recently reported technique to use Pluronic F127 as a thermally switchable encapsulant for *C*. *elegans* has the opportunity to expand the breadth of operations that can be performed by *in situ* photopatterning, though material properties and compatibility must be considered with respect to the specific aims of each study [4].

In conclusion, we have presented a system and method for rapid microfabrication of hydrogel-based microstructures, with direct compatibility with standard NGM substrates used to culture *C*. *elegans*. The utility of this method was validated by performing example assays requiring direct interaction from the researcher. In the future, dynamic photopatterning can be used in conjunction with machine learning and automated image analysis techniques. This would enable large data sets to be acquired and processed in an automated fashion, accelerating breakthroughs in understanding the behavior of model organisms such as *C*. *elegans*, in response to dynamic geometric environments.

## Supporting Information

S1 Data Raw tabular data. The raw data collected in the experiments shown is provided in tabular form.
(XLSX)

S1 Fig Overview of the maskless lithography system. **(a)** Schematic of the UV DLP optofluidic lithography system. **(b)** An overlay of the optical trains discussed with the schematic.
(TIF)

S2 Fig Chemical structure of PEG-DA. (TIF)

S3 Fig ATR-FTIR results during PEG-DA diffusion into NGM. **(a)** ATR-FTIR spectra of NGM taken at sequential times after placing a drop of PEG-DA on top of a thin slab (200 μm) and measuring from the bottom. **(b)** Emergence of ester carbonyl band from PEG indicates PEG-DA diffuses into NGM, highlighting area of red box in **(a)**.
(TIF)

S4 Fig Photographs of *C*. *elegans* observed after exposure to 20% PEG-DA, which were studied for signs of morphological changes. **(a)** Time = 0 min. **(b)** Time = 5 min. **(c)** Time = 10 min. **(d)** Time = 20 min. Peak velocity slowed over 20 minutes but increased again after a period of rest.
(TIF)

S5 Fig *C*. *elegans* viability comparing NGM gel substrate and PEG-DA/NGM substrate. (TIF)

S1 File The supporting information further discusses the design and parts necessary to construct this system, PEG-DA formulation selection, ATR-FTIR analysis details and survival data of the nematodes. (DOCX)

S1 Programs Programs for printing and analysis. Three programs are provided in this zip file including a labview interface (On-demand photopatterning Labview) to control the projection system, a matlab file to analyze the ATR_FTIR data provided (ATR_FTIR_*C_elegans*_Videos.m) and a python software script needed to track worms from the video file (*C_elegans*_video_tracking.py).
(ZIP)

S1 STL 3D printing file for DMD housting. (STL)

S2 STL 3D printing file for DMD housing lid. (STL)

S1 Video Worm swimming among pillars. (MP4)

S2 Video Worm interacting with free floating pin. (MP4)

S3 Video Researcher drawing with a tablet. (8x). (MP4)

S4 Video Worm solving a maze. (MP4)

## References

[1] Park S, Hwang H, Nam S-W, Martinez F, Austin RH, Ryu WS. Enhanced Caenorhabditis elegans locomotion in a structured microfluidic environment. Brembs B, editor. PLoS One [Internet]. Public Library of Science; 2008 1 [cited 2014 Oct 24];3(6):e2550 Available from: http://dx.plos.org/10.1371/journal.pone.0002550 10.1371/journal.pone.0002550 18575618 PMC2430527

[2] Qin J, Wheeler AR. Maze exploration and learning in C. elegans. Lab Chip [Internet]. The Royal Society of Chemistry; 2007 2 31 [cited 2014 Oct 19];7(2):186–92. Available from: http://pubs.rsc.org/en/content/articlehtml/2007/lc/b613414a 17268620 10.1039/b613414a

[3] Eisenmann DM. WormBook. WormBook [Internet]. 2005 1 [cited 2014 Nov 11];1–17. Available from: http://www.ncbi.nlm.nih.gov/pubmed/18050402

[4] Hwang H, Krajniak J, Matsunaga Y, Benian GM, Lu H. On-demand optical immobilization of Caenorhabditis elegans for high-resolution imaging and microinjection. Lab Chip [Internet]. Royal Society of Chemistry; 2014 9 21 [cited 2014 Dec 4];14(18):3498–501. Available from: http://www.ncbi.nlm.nih.gov/pubmed/25056343 10.1039/c4lc00697f 25056343 PMC4148454

[5] Chronis N. Worm chips: microtools for C. elegans biology. Lab Chip [Internet]. The Royal Society of Chemistry; 2010 2 21 [cited 2014 Oct 24];10(4):432–7. Available from: http://pubs.rsc.org/en/content/articlehtml/2010/lc/b919983g 10.1039/b919983g 20126682

[6] Lee SA, Chung SE, Park W, Lee SH, Kwon S. Three-dimensional fabrication of heterogeneous microstructures using soft membrane deformation and optofluidic maskless lithography. Lab Chip [Internet]. 2009 6 21 [cited 2011 Jul 25];9(12):1670–5. Available from: http://www.ncbi.nlm.nih.gov/pubmed/19495448 10.1039/b819999j 19495448

[7] Au AK, Lee W, Folch A. Mail-order microfluidics: evaluation of stereolithography for the production of microfluidic devices. Lab Chip [Internet]. 2014 4 7 [cited 2014 Jul 11];14(7):1294–301. Available from: http://www.ncbi.nlm.nih.gov/pubmed/24510161 10.1039/c3lc51360b 24510161 PMC4362723

[8] Totsu K, Fujishiro K, Tanaka S, Esashi M. Fabrication of three-dimensional microstructure using maskless gray-scale lithography. Sensors Actuators, A Phys. 2006;130-131(SPEC. ISS.):387–92.

[9] Curley JL, Jennings SR, Moore MJ. Fabrication of Micropatterned Hydrogels for Neural Culture Systems using Dynamic Mask Projection Photolithography. J Vis Exp. 2011;(48). 10.3791/2636 PMC3197419 21372777

[10] Arcaute K, Mann BK, Wicker RB. Stereolithography of three-dimensional bioactive poly(ethylene glycol) constructs with encapsulated cells. Ann Biomed Eng [Internet]. 2006 9 [cited 2014 Oct 22];34(9):1429–41. Available from: http://www.ncbi.nlm.nih.gov/pubmed/16897421 16897421 10.1007/s10439-006-9156-y

[11] Chung SE, Park W, Park H, Yu K, Park N, Kwon S. Optofluidic maskless lithography system for real-time synthesis of photopolymerized microstructures in microfluidic channels. Appl Phys Lett [Internet]. 2007 [cited 2011 Jul 17];91(4):041106 Available from: http://link.aip.org/link/APPLAB/v91/i4/p041106/s1&Agg=doi

[12] Seol YJ, Park DY, Park JY, Kim SW, Park SJ, Cho DW. A new method of fabricating robust freeform 3D ceramic scaffolds for bone tissue regeneration. Biotechnol Bioeng. 2013;110(5):1444–55. 10.1002/bit.24794 23192318

[13] Brenner S. The genetics of Caenorhabditis elegans. Genetics [Internet]. 1974 5 [cited 2014 Jul 18];77(1):71–94. Available from: http://www.pubmedcentral.nih.gov/articlerender.fcgi?artid=1213120&tool=pmcentrez&rendertype=abstract 4366476 10.1093/genetics/77.1.71 PMC1213120

[14] Bahney CS, Lujan TJ, Hsu CW, Bottlang M, West JL, Johnstone B. Visible light photoinitiation of mesenchymal stem cell-laden bioresponsive hydrogels. Eur Cells Mater. 2011;22:43–55. 10.22203/ecm.v022a04 PMC5050040 21761391

[15] Silverstein R. Spectrometric identification of organic compounds. New York: Wiley; 1981.

[16] Wu H, Odom TW, Chiu DT, Whitesides GM. Fabrication of Complex Three-Dimensional Microchannel Systems in PDMS. 2003;(19):554–9. 10.1021/ja021045y 12517171

[17] Mata A, Fleischman AJ. Characterization of Polydimethylsiloxane (PDMS) Properties for Biomedical Micro / Nanosystems. Exposure. 2005;2:281–93. 10.1007/s10544-005-6070-2 16404506

[18] Spotts JM. Fabrication of PDMS Microfluidic Devices Have fun, ask questions and play ! Syst Biol (Stevenage). 2008;

[19] Cuchiara MP, Allen ACB, Chen TM, Miller JS, West JL. Multilayer microfluidic PEGDA hydrogels. Biomaterials [Internet]. 2010 7 [cited 2014 Oct 24];31(21):5491–7. Available from: http://www.ncbi.nlm.nih.gov/pubmed/20447685 10.1016/j.biomaterials.2010.03.031 20447685

[20] Jung YS, Chang JB, Verploegen E, Berggren KK, Ross C a. A path to ultranarrow patterns using self-assembled lithography. Nano Lett [Internet]. 2010 3 10 [cited 2011 Jul 26];10(3):1000–5. Available from: http://www.ncbi.nlm.nih.gov/pubmed/20146429 10.1021/nl904141r 20146429

[21] Lee D-H. Optical System with 4 ㎛ Resolution for Maskless Lithography Using Digital Micromirror Device. J Opt Soc Korea [Internet]. 2010 9 25 [cited 2014 Oct 24];14(3):266–76. Available from: http://koreascience.or.kr/journal/view.jsp?kj=E1OSAB&py=2010&vnc=v14n3&sp=266

[22] Hansotte EJ, Carignan EC, Meisburger WD. High speed maskless lithography of printed circuit boards using digital micromirrors.

[23] Chan KF. High-resolution maskless lithography. J Micro/Nanolithography, MEMS, MOEMS [Internet]. 2003 10 1 [cited 2014 Oct 19];2(4):331 Available from: http://nanolithography.spiedigitallibrary.org/article.aspx?doi=10.1117/1.1611182

[24] Lake J, Walsh K, Mcnamara S. Low-cost maskless grayscale lithography using a new photo-definable polymide for polymer mems applications. 2009;100:1889–91.

[25] Krajniak J, Lu H. Long-term high-resolution imaging and culture of C. elegans in chip-gel hybrid microfluidic device for developmental studies. Lab Chip [Internet]. The Royal Society of Chemistry; 2010 7 21 [cited 2014 Oct 24];10(14):1862–8. Available from: http://pubs.rsc.org/en/content/articlehtml/2010/lc/c001986k 10.1039/c001986k 20461264 PMC8102136

[26] Dendukuri D, Panda P, Haghgooie R, Kim JM, Hatton TA, Doyle PS. Modeling of Oxygen-Inhibited Free Radical Photopolymerization in a PDMS Microfluidic Device. Macromolecules [Internet]. 2008 11 25 [cited 2011 Jun 26];41(22):8547–56. Available from: http://pubs.acs.org/doi/abs/10.1021/ma801219w

[27] Lockery SR, Lawton KJ, Doll JC, Faumont S, Coulthard SM, Thiele TR, et al Artificial dirt: microfluidic substrates for nematode neurobiology and behavior. J Neurophysiol [Internet]. 2008 6 [cited 2014 Oct 24];99(6):3136–43. Available from: http://www.pubmedcentral.nih.gov/articlerender.fcgi?artid=2693186&tool=pmcentrez&rendertype=abstract 10.1152/jn.91327.2007 18337372 PMC2693186

[28] Majmudar T, Keaveny EE, Zhang J, Shelley MJ. Experiments and theory of undulatory locomotion in a simple structured medium. J R Soc Interface [Internet]. 2012 8 7 [cited 2014 Oct 24];9(73):1809–23. Available from: http://www.pubmedcentral.nih.gov/articlerender.fcgi?artid=3385758&tool=pmcentrez&rendertype=abstract 10.1098/rsif.2011.0856 22319110 PMC3385758

[29] Kim J, Chung SE, Choi S-E, Lee H, Kim J, Kwon S. Programming magnetic anisotropy in polymeric microactuators. Nat Mater [Internet]. Nature Publishing Group; 2011 10 [cited 2015 Jan 22];10(10):747–52. Available from: http://www.ncbi.nlm.nih.gov/pubmed/21822261 10.1038/nmat3090 21822261

[30] Pandey S, Joseph A, Lycke R, Parashar A. Decision-making by nematodes in complex microfluidic mazes. Adv Biosci Biotechnol [Internet]. Scientific Research Publishing; 2011 12 2 [cited 2014 Feb 7];02(06):409–15. Available from: http://www.scirp.org/journal/PaperInformation.aspx?PaperID=8897&#abstract

[31] Hulme SE, Shevkoplyas SS, McGuigan AP, Apfeld J, Fontana W, Whitesides GM. Lifespan-on-a-chip: microfluidic chambers for performing lifelong observation of C. elegans. Lab Chip [Internet]. 2010 3 7 [cited 2014 Oct 2];10(5):589–97. Available from: http://www.pubmedcentral.nih.gov/articlerender.fcgi?artid=3060707&tool=pmcentrez&rendertype=abstract 10.1039/b919265d 20162234 PMC3060707

[32] Chronis N, Zimmer M, Bargmann CI. Microfluidics for in vivo imaging of neuronal and behavioral activity in Caenorhabditis elegans. Nat Methods [Internet]. 2007 9 [cited 2014 Dec 10];4(9):727–31. Available from: http://www.ncbi.nlm.nih.gov/pubmed/17704783 17704783 10.1038/nmeth1075

[33] Mondal S, Ahlawat S, Koushika SP. Simple microfluidic devices for in vivo imaging of C. elegans, Drosophila and zebrafish. J Vis Exp [Internet]. 2012 1 [cited 2014 Dec 10];(67). Available from: http://www.pubmedcentral.nih.gov/articlerender.fcgi?artid=3490237&tool=pmcentrez&rendertype=abstract 10.3791/3780 PMC3490237 23051668

