# Supplementary figures and images for: On-Demand Isolation and Manipulation of C. elegans by In Vitro Maskless Photopatterning
Source: PLoS One. 2016 Jan 5;11(1):e0145935. doi: 10.1371/journal.pone.0145935 (PMC4701667; doi:10.1371/journal.pone.0145935)

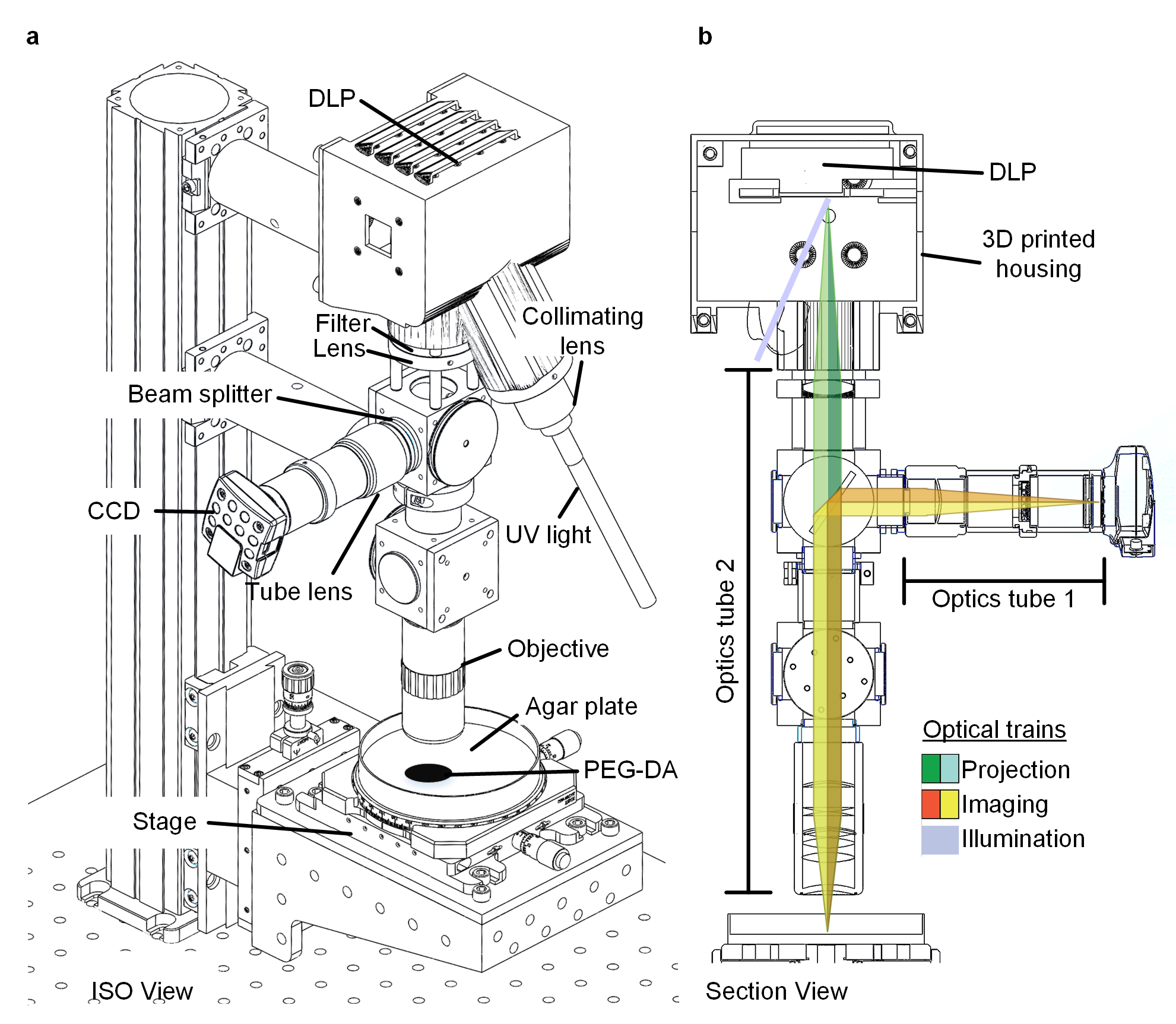

Supplement: S1 Fig — (a) Schematic of the UV DLP optofluidic lithography system. (b) An overlay of the optical trains discussed with the schematic. (TIF) [file pone.0145935.s002.tif]

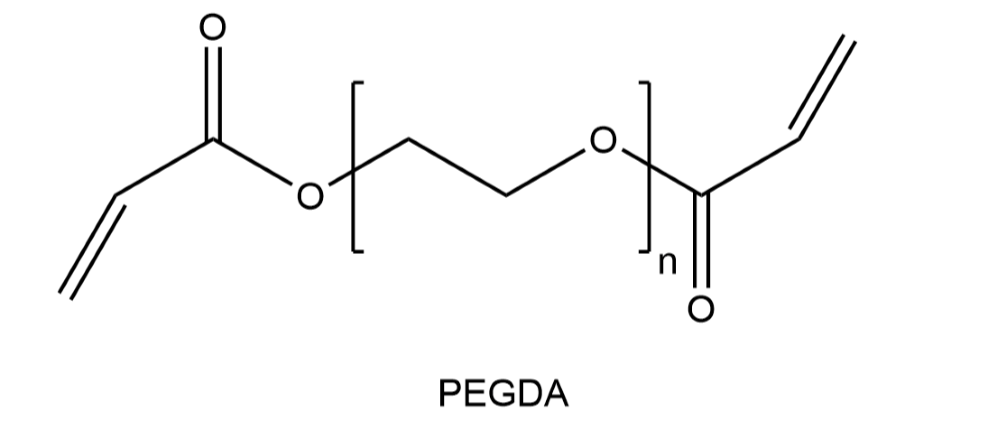

Supplement: S2 Fig — (TIF) [file pone.0145935.s003.tif]

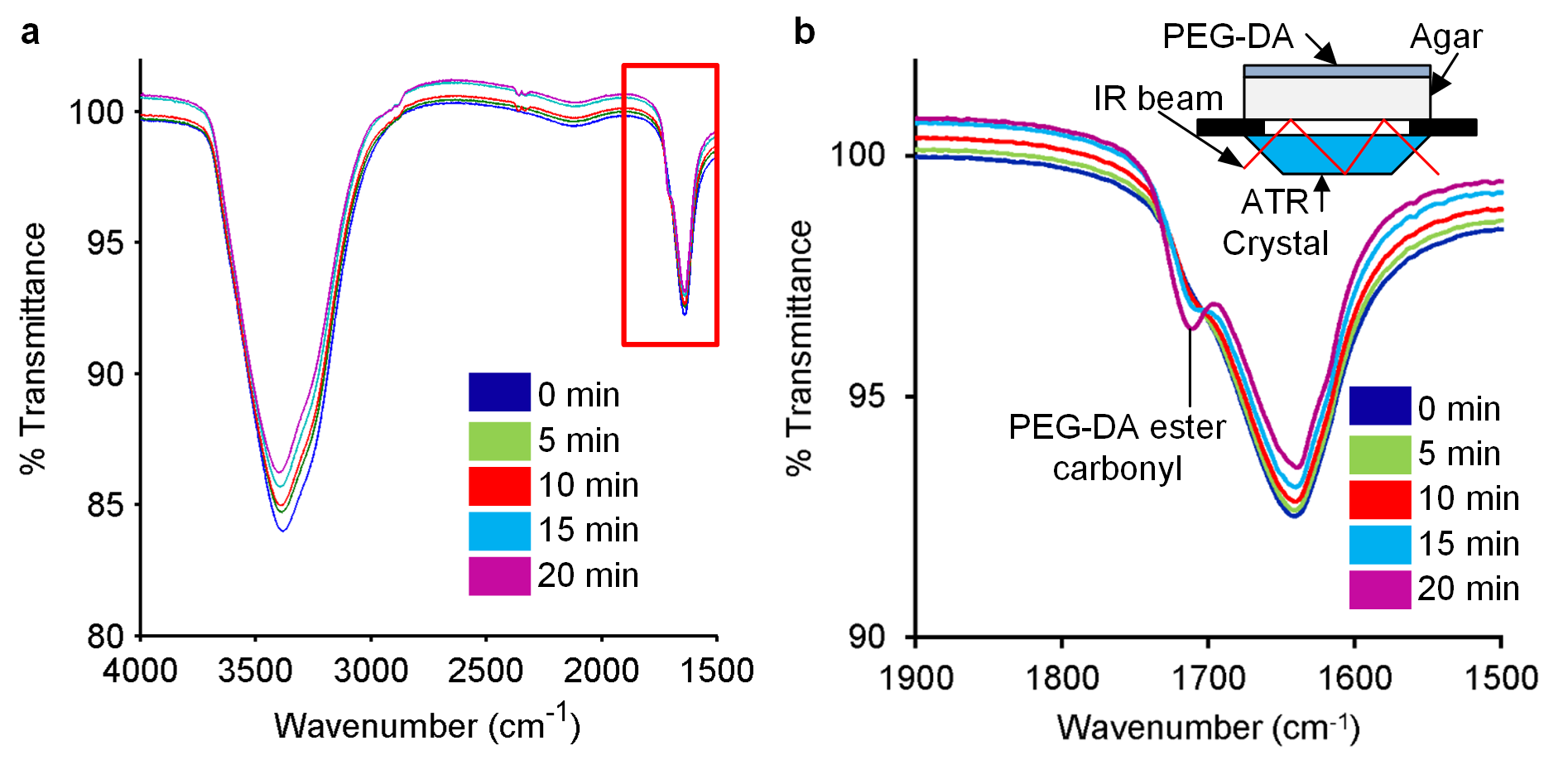

Supplement: S3 Fig — (a) ATR-FTIR spectra of NGM taken at sequential times after placing a drop of PEG-DA on top of a thin slab (200 μm) and measuring from the bottom. (b) Emergence of ester carbonyl band from PEG indicates PEG-DA diffuses into NGM, highlighting area of red box in (a). (TIF) [file pone.0145935.s004.tif]

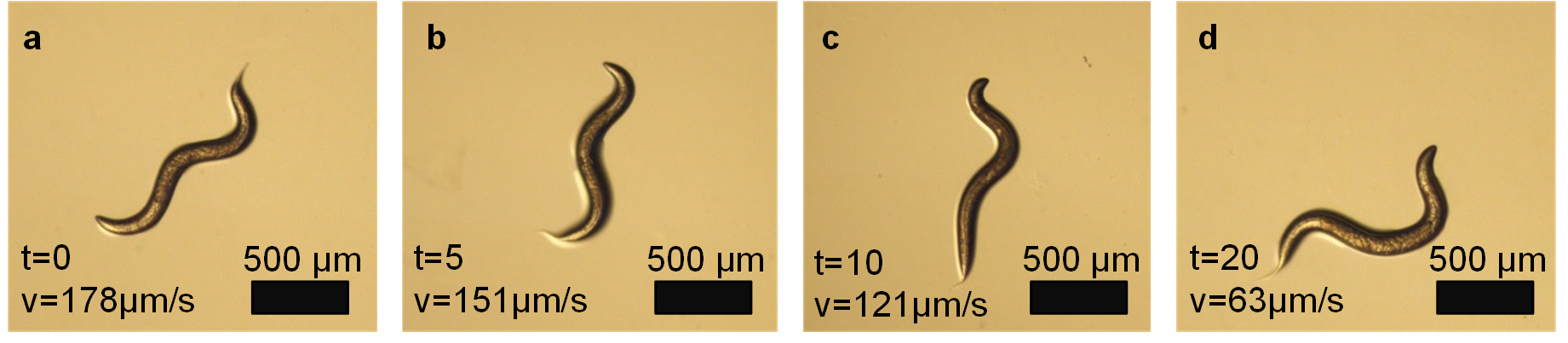

Supplement: S4 Fig — (a) Time = 0 min. (b) Time = 5 min. (c) Time = 10 min. (d) Time = 20 min. Peak velocity slowed over 20 minutes but increased again after a period of rest. (TIF) [file pone.0145935.s005.tif]

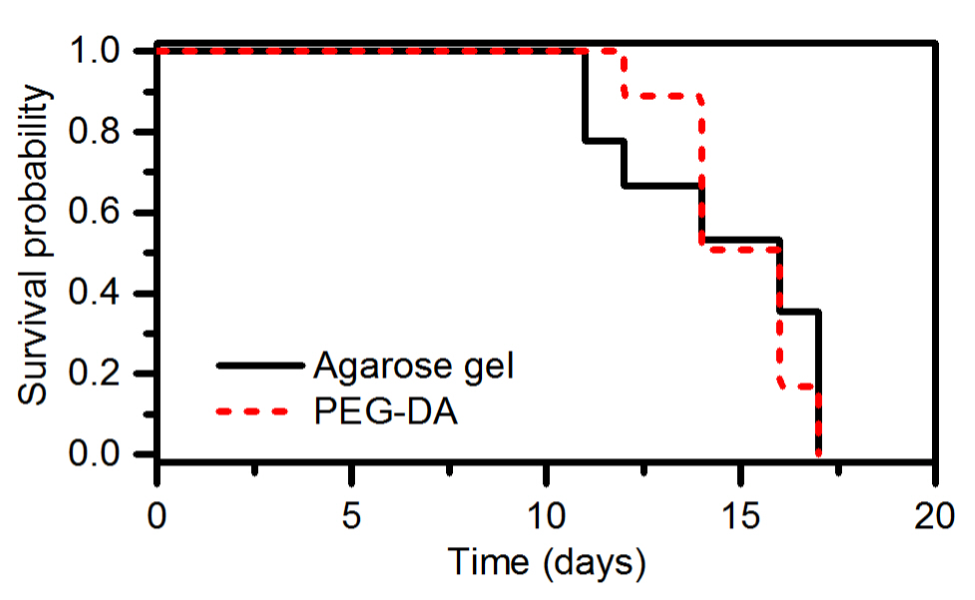

Supplement: S5 Fig — (TIF) [file pone.0145935.s006.tif]
