# Supplementary material for: On-Demand Isolation and Manipulation of C. elegans by In Vitro Maskless Photopatterning
Source: PLoS One. 2016 Jan 5;11(1):e0145935. doi: 10.1371/journal.pone.0145935 (PMC4701667; doi:10.1371/journal.pone.0145935)
Supplement: S1 File — (DOCX) [file pone.0145935.s007.docx]

**SUPPORTING INFORMATION for**

**On-demand isolation and manipulation of *C. elegans* by *in vitro* maskless photopatterning.**

C. Ryan Oliver^1,2^, Eleni Gourgou^1^, Daphne Bazopoulou^1^, Nikos Chronis^1,3^, and A. John Hart^2^*

^1^Department of Mechanical Engineering, University of Michigan, Ann Arbor, MI. 48109, USA

^2^Department of Mechanical Engineering and Laboratory for Manufacturing and Productivity, Massachusetts Institute of Technology, Cambridge, MA 02139, USA

^3^Department of Biomedical Engineering, University of Michigan, Ann Arbor, MI. 48109, USA

*Corresponding author: [ajhart@mit.edu](mailto:ajohnh@umich.edu), 617.324.7022

**System design**

As shown in S1 Fig the design of the system and layout is made of three lens tubes. As depicted a second optional beam splitter can be used for inline lighting rather than lighting from below as was done for the experiments presented. The 3D printed housing properly aligns and positions the DMD, a total internal reflectance prism (TIR), the fiber light input and the microscope projection tube. We provide the list of materials necessary to construct the system in Table A and the solid models as attachments.

**Table A.** Components of maskless photopatterning system shown in S1 Fig.

| Vendor + contact | Part# | Details | Qty |
| --- | --- | --- | --- |
| Electronics |  |  |  |
| Nikon | D5100 | 24Mp DSLR | 1 |
| Dymax | 75 | BlueWave 75 UV light source | 1 |
| Dymax | 5mmx1.5m | 5 mm Lightguide | 1 |
| Wintech | W4100 | UV DLP XGA | 1 |
| SainSmart | 20-018-101 | 4-Channel 5V relay board | 1 |
| National Instruments | USB-6008 | USB DAQ | 1 |
| Support Structures/Optomechanics | | |  |
| McMaster-Carr | 70085K24 | Wire wrap | 1 |
| Automation Direct | HW-161408CHQR | NEMA enclosure | 1 |
| Automation Direct | HW-MP1614A | Sub panel aluminum | 1 |
| Automation Direct | HW-MGFTKIT | Mounting foot kit | 1 |
| Adorama | ARM | Novoflex goosneck 17.7" | 1 |
| Edmundoptics | NT59-333 | 6" Travel long travel rack and pinion track | 1 |
| Thorlabs | PBH11106 | Optical breadboard 3'x2' | 1 |
| Thorlabs | OT1 | Objective turret | 1 |
| Thorlabs | AV2 | Vibration isolation feet | 1 |
| Thorlabs | XYR1 | XY A stage | 1 |
| Thorlabs | XT66-500 | 500mm long 66mm optics rail | 1 |
| Thorlabs | P8 | 1.5" Post 8" long | 4 |
| Thorlabs | XT66P2 | XT66 Rail carriage | 2 |
| Thorlabs | XT66P1 | XT66 Vertical mounting plate | 1 |
| Thorlabs | PH1 | 1" Post holder | 2 |
| Thorlabs | TR075 | 1/2" Post x3/4" long | 2 |
| Thorlabs | PT1A | 1" Single axis stage | 1 |
| Thorlabs | AP90RL | Right angle bracket large | 1 |
| Thorlabs | XT66C4 | Clamping platform for 66 mm rail | 2 |
| Thorlabs | C1520 | Adapter plate for post mounting clamp | 3 |
| Thorlabs | SM1TC | SM1 Clamp | 2 |
| Thorlabs | P4 | 1.5" Mounting post L=4" | 6 |
| Thorlabs | VB01 | Vertical bracket for breadboards | 2 |
| *Optics tube 1* |  |  |  |
| Thorlabs | SM1L10 | SM1 1" Tube | 1 |
| Thorlabs | SM1A39 | C to SM1 | 3 |
| Thorlabs | SM1L40 | Sm1 4" Tube | 2 |
| Thorlabs | SM1L05 | SM1 0.5" Tube | 1 |
| Thorlabs | CFW6 | 30mm Cage filter wheel 1" | 1 |
| Thorlabs | SM1T2 | 1" to 1" interal thread adapter | 2 |
| Edmund Optics | MT-L4 | Mitutoyo 200 mm tube lens | 1 |
| Edmund Optics | 66-027 | Mitutoyo 200 mm tube lens holder | 1 |
| Edmund Optics | 54-341 | C to F Mount adapter | 1 |
| *Optics tube 2* |  |  |  |
| Thorlabs | SM1A3TS | SM1 to RMS objective adapter | 1 |
| Thorlabs | SM1L03 | SM1 Lens tube, L = 0.3" | 1 |
| Thorlabs | FGUV | UV Filter 1" | 1 |
| Thorlabs | LBF254-150 | 150 mm lens | 1 |
| Thorlabs | CP70T | 30 mm cage plate | 4 |
| Thorlabs | ER4 | 4 inch long cage rods | 8 |
| Thorlabs | ACL7560 | Collimating aspherical lens | 1 |
| Thorlabs | AD8F | Fiber adapter to SM1 | 1 |
| Thorlabs | WG41010 | Glass window 1" diameter | 1 |
| Thorlabs | SM1T2 | 1" to 1" internal thread adapter | 2 |
| Thorlabs | DMLP505R | 1" Longpass Dichroic Mirror Beam Splitter | 1 |
| Thorlabs | B5C | Beam splitter mount | 2 |
| Thorlabs | B3C | Beam splitter rotation | 4 |
| Thorlabs | C4W | Beam splitter block | 2 |
| Thorlabs | SM1CP2 | Beam splitter block covers | 6 |
| Thorlabs | EPB1 | 1" Longpass dichroic mirror beam splitter | 1 |
| Edmund Optics | 46-404 | Mitutoyo 20X plan Apo objective | 1 |
| Edmund Optics | 46-143 | Mitutoyo 5X plan Apo objective | 1 |
| Edmund Optics | 58-296 | Mitutoyo to RMS adapter | 2 |
| Custom | 1 | 3D printed housing (**S1 and S2 STL**) | 1 |

**Selection of the PEG-DA formulation**

The chemical structures of PEG-DA are shown in S2 Fig. The PEG-DA monomers cross-link during UV light exposure by radical based photo-polymerization. To determine a suitable photoinitator for use with live *C. elegans* culture, we tested three candidate photoinitators, 2-hydroxy-2methyl-1-phenyl-1-propanone (Darocur 1173), Phenylbis (2,4,6-trimethylbenzoyl)-phosphine oxide and a custom mixture of 0.01 mM Eosin Y and 0.1% TEA (Triethanolamine). As documented in Table B, the latter two initiate polymerization in white light while the former initiates polymerization in NUV (near visible ultraviolet light). We prepared various concentrations of Phenylbis (2,4,6-trimethylbenzoyl)-phosphine oxide in PEG-DA by titration and found no combinations of the ingredients would both photopolymerize and remain biocompatible. The Eosin Y solution would not adhere to the NGM after photocuring for 5 minutes but did show promise as a biocompatible photopolymer with *C. elegans*. Finally, the NUV Photoinitiator (Darocur 1173) mixed with PEG-DA showed the ability to both adhere and be compatible with living *C. elegans* based on the survival assay results. Based on the above results, we chose to proceed using Darocur 1173.

The data in this section was measured by first testing biocompatability as described in the methods section in which a drop was placed on NGM plates seeded with worms and observed for ten minutes. This was done for each photoinitiator at four levels and at four levels of buffer concentrations (0%, 50%, 75%, 88%). If there was no observable shock or mortality then the sample was exposed to the appropriate wavelength and observed until polymerization occurred for a maximum of five minutes. Longer exposures may have resulted in polymerization but were not amenable to this technique because the system should respond quickly to worm motion. Finally, a plate was exposed to the resolution test pattern and rinsed to observe adhesion. Rinsing was done using buffer in a syringe dispensed across the surface of the plate while the plate is held at a 45˚ angle. If features remained that were smaller than 100 μm then it was noted as a “Yes” in the table. The size was chosen because it is on the scale of young *C. elegans* and 10 times smaller than adult *C. elegans*.

**Table B**. Results of testing biocompatibility and adherence to NGM plates of three photoinitiators. Biocompatibility was tested as described in Methods. The selected photoinitiator is Darocur 1173. N/A: not tested, because compound was not biocompatible. NUV: Near ultraviolet light (365 nm).

|  | 2-hydroxy-2methyl-1-phenyl-1-propanone (Darocur 1173) | Phenylbis (2,4,6-trimethylbenzoyl)-phosphine oxide | 0.01 mM Eosin Y and 0.1% TEA |
| --- | --- | --- | --- |
| Polymerization | NUV | White light | White light |
| Adhesion | Yes | N/A | No |
| Biocompatibility | Yes | No | Yes |

**Adhesion analysis via ATR-FTIR**

Diffusion of the PEG-DA polymer into the NGM was measured by ATR-FTIR. A baseline spectrum was taken and measured every 5 minutes for a total duration of 20 minutes, as shown in S3 Fig. In S3 Fig, a comparison between the first and last measurement is presented, showing the development of a band (at 1714 cm^-1^) due to the presence of an ester carbonyl. We interpret this band as an indication of diffusion, because this group can only be found in the PEG-DA monomer. To facilitate this measurement, a drop (10 μl) of PEG-DA is placed on the surface of a thin 0.2 mm slab of NGM which is placed on top of the ATR crystal. When sampling by ATR-FTIR, the infrared beam is reflected at the interface of the crystal and the sample. In our experiment, the refractive index of the sample is lower than the crystal (4) because the agar is composed of a large proportion of water (1.3). As a result, evanescent waves will form at the reflection points that penetrate into the sample, and this depth will be restricted to 0.5-10 μm with the value dependent on the wavelength of light, the angle of incidence and the refractive of the crystal and NGM. Therefore, in order to be detected, the PEG-DA must diffuse at least 190 μm into the NGM (from the opposite side as the measurement). We hypothesize that this diffusion creates a stronger joint upon photopolymerization due to the diffusive interface between the PEG-DA and NGM.

**Survival of *C. elegans***

Before performing live worm assays using the dynamic lithography method, it was essential to evaluate the viability of the worms in the presence of the PEG-DA. The viability test was performed on NGM plates non-seeded with *Escherichia coli* OP50. Each plate was seeded with adult worms of various ages and a droplet of the material under investigation was placed onto them. We incubated the worms within the PEG-DA solution droplet at 20 ᵒC and checked on the worms every 1 min for 20 min.

We found that a solution consisting of 20% PEG-DA and 79.5% water with 0.5% Darocur 1173 does not influence *C. elegans’* survival (S4 Fig). No morphological changes or dehydration were observed during the observation period from time 0-20 minutes for a set of 10 worms and 2 independent experiments. *C. elegans* motility and pharyngeal pumping after exposure to the 20% PEG-DA formulation resulted in no observable change from control at 5, 10 and 20 minutes. This was checked by observing pharyngeal pumping, stroking with a platinum wire and by tapping the side of the NGM plate.

Greater concentrations of PEG-DA, 30%, 50%, 75% and 100% appear to induce stress on worms, expressed as reduced motility and response to poking, increased curling and signs of dehydration, resulting in death after 3-10 minutes. These results, in combination with the fact that the suitable photo-initiator was found to be insoluble at PEG-DA concentrations below 15%, led us to choose 20% PEG-DA as the appropriate concentration for dynamic lithography of *C. elegans*. To support our observation showing no apparent effect of 20% PEG-DA in *C. elegans* short-term viability, we performed a survival assay to verify that this is also the case with the worm’s lifespan. For this, we used NGM plates, seeded with *Escherichia coli* OP50, rinsed in PEG-DA using a syringe, on which we built simple structures from photopolymerized PEG-DA (i.e. rings, rectangular frames). We then placed worms on the plate, as described in Methods. As shown in S5 Fig, the presence of PEG-DA has no effect on long-term viability of *C. elegans*. The worms studied in this experiment also showed normal locomotion patterns and motility, no signs of dehydration, and a normal life cycle. These results encouraged us to use 20% PEG-DA and instilled confidence this was a suitable platform for *in situ* culture with photopatterning.

**Supplementary videos**

**Table C.** Supplementary videos

| Video | Description |
| --- | --- |
| S1 Video | Worm swimming among pillars |
| S2 Video | Worm interacting with free floating pin |
| S3 Video | Researcher drawing with a tablet (8x) |
| S4 Video | Worm solving a maze |
